# Bumblebee colony development following chronic exposure to field-realistic levels of the neonicotinoid pesticide thiamethoxam under laboratory conditions

**DOI:** 10.1038/s41598-017-08752-x

**Published:** 2017-08-14

**Authors:** Dara A. Stanley, Nigel E. Raine

**Affiliations:** 1 School of Biological Sciences, Royal Holloway University of London, Egham, TW20 0EX UK; 2 Botany and Plant Science, School of Natural Sciences and Ryan Institute, National University of Ireland Galway, Galway, Ireland; 3 School of Environmental Sciences, University of Guelph, Guelph, Ontario, N1G 2W1 Canada

## Abstract

Neonicotinoid pesticides are used in agriculture to reduce damage from crop pests. However, beneficial insects such as bees can come into contact with these pesticides when foraging in treated areas, with potential consequences for bee declines and pollination service delivery. Honeybees are typically used as a model organism to investigate insecticide impacts on bees, but relatively little is known about impacts on other taxa such as bumblebees. In this experiment, we chronically exposed whole mature bumblebee (*Bombus terrestris*) colonies to field-realistic levels of the neonicotinoid thiamethoxam (2.4ppb & 10ppb) over four weeks, and compared colony growth under laboratory conditions. We found no impact of insecticide exposure on colony weight gain, or the number or mass of sexuals produced, although colonies exposed to 2.4ppb produced larger males. As previous studies have reported pesticide effects on bumblebee colony growth, this may suggest that impacts on bumblebee colonies are more pronounced for colonies at an earlier stage in the reproductive cycle. Alternatively, it may also indicate that thiamethoxam differs in toxicity compared to previously tested neonicotinoids in terms of reproductive effects. In either case, assessing bumblebee colony development under field conditions is likely more informative for real world scenarios than tests conducted in laboratory conditions.

## Introduction

Bumblebees are important pollinators in temperate regions, providing essential pollination services to both crops and wild plants^1–3^. However, concern over the decline of bumblebee pollinators^4–10^ has resulted in recent interest in the potential role of pesticide exposure as one of the factors driving these declines. Bumblebees foraging in agricultural environments are likely to encounter a range of pesticides applied to crops as sprays, soil drenches or seed treatments. The dramatic rise in use of systemic neonicotinoid seed treatments, now the world’s most widely used class of insecticides^11^, raise particular concerns as the pesticide active ingredients are translocated into all tissues of the treated crop, including the nectar and pollen that can be collected and consumed by bees^12–14^. In addition, as neonicotinoid insecticides are also found in soils^15^ and water courses^16, 17^, it is perhaps unsurprising that residues have also been detected in wild flowering plants^18–22^. Therefore, there are multiple possible routes of exposure for bumblebees to these insecticides over extended periods of time, resulting in chronic exposure. These levels of exposure in the field are typically below levels that cause direct mortality in bees, but can cause a range of sub lethal effects^23–25^.

However, while risk assessments for pesticide registration and usage are currently carried out using honeybees as the only model for insect pollinators^24–27^, impacts on bumblebees and other taxa can be different^28–32^. In the last few years, a greater number of studies reporting impacts of exposure to neonicotinoid insecticides on bumblebees have been published, most of which have investigated the effects of imidacloprid^26^. Indeed, exposure to field-realistic levels of imidacloprid have been shown to affect bumblebee foraging^33, 34^, colony growth and reproduction^34–37^. Imidacloprid use is declining, and other neonicotinoids (e.g. thiamethoxam and clothianidin) are being used widely as seed treatments for oilseed rape (canola), maize (corn), soy and other crops worldwide^11, 16, 38^. In comparison to imidacloprid, less is known about the impacts of exposure to thiamethoxam and clothianidin on bumblebees (but see refs 26, 31, 39–48).

The aim of our experiment was to investigate potential impacts of thiamethoxam on bumblebee (*Bombus terrestris*) colony growth in a laboratory setting. We exposed bumblebee colonies chronically to low, field-realistic levels of thiamethoxam in sugar water solutions for a period of up to 27 days, to mimic exposure through nectar while foraging on treated crops such as oilseed rape (*Brassica napus*). Eight established colonies were exposed to an untreated sucrose solution (control treatment) and eight colonies each were exposed to a sucrose solution containing either 2.4ppb and 10ppb thiamethoxam respectively. After a further 13–14 days we counted the numbers of workers and sexuals produced, and estimated the average weight of individuals and total biomass of each caste per colony.

## Results

On average, colonies gained 336 g in weight over the course of the experiment, and produced 389 workers (range 146–606) and 261 males (range 132–397; see Table S1). Males were on average bigger than workers (mean dry mass: males 0.09 g, workers 0.05 g). Seven of 24 colonies produced queens (2 colonies each from control and 10ppb treatments, and 3 from the 2.4ppb treatment group), and numbers of queens produced ranged from two to 34 per colony. There were no differences in colony weight among treatments at the start of the experiment (F_2,21_ = 0.014, p = 0.99) or the number of individuals they contained (F_2,21_ = 1.74, p = 0.2; Table 1). However, colony weight did not correlate with the numbers of individuals in each colony (Pearsons product moment correlation; t = 1.3, df = 22, p = 0.22).

**Table 1:** Means (±standard error) of the variables measured per *Bombus terrestris* colony. N = eight colonies per treatment (24 colonies in total).

		control	2.4ppb	10ppb
Colony weights				
Colony weight (start, g)		549.1 ± 6.7	549.3 ± 6.6	550.5 ± 5.8
No. workers (start)		94 ± 4.3	95 ± 7.6	108 ± 6.0
Weight change (g) over experiment		336.56 ± 11.1	348.99 ± 17.9	322.46 ± 9.3
Total number bees produced over experiment				
No. workers		375 ± 43.45	400 ± 25.41	393 ± 51.73
No. males		275 ± 20.83	231 ± 29.04	279 ± 28.91
No. queens		1.75 ± 0.53	8.4 ± 4.21	2.25 ± 1.11
Dry weight of individuals produced				
Average worker weight (g)		0.052 ± 0.003	0.057 ± 0.004	0.052 ± 0.002
Average male weight (g)		0.083 ± 0.003	0.104 ± 0.008	0.086 ± 0.003
Average queen weight (g)		0.25 ± 0.020	0.25 ± 0.013	0.24 ± 0.015
Total biomass produced				
Total worker biomass (g)		19.43 ± 2.32	22.19 ± 0.87	19.54 ± 1.97
Total male biomass (g)		22.99 ± 2.12	23.53 ± 3.14	23.95 ± 2.63
Total queen biomass (g)		0.37 ± 0.06	1.90 ± 0.98	0.46 ± 0.18

**Figure 1:**
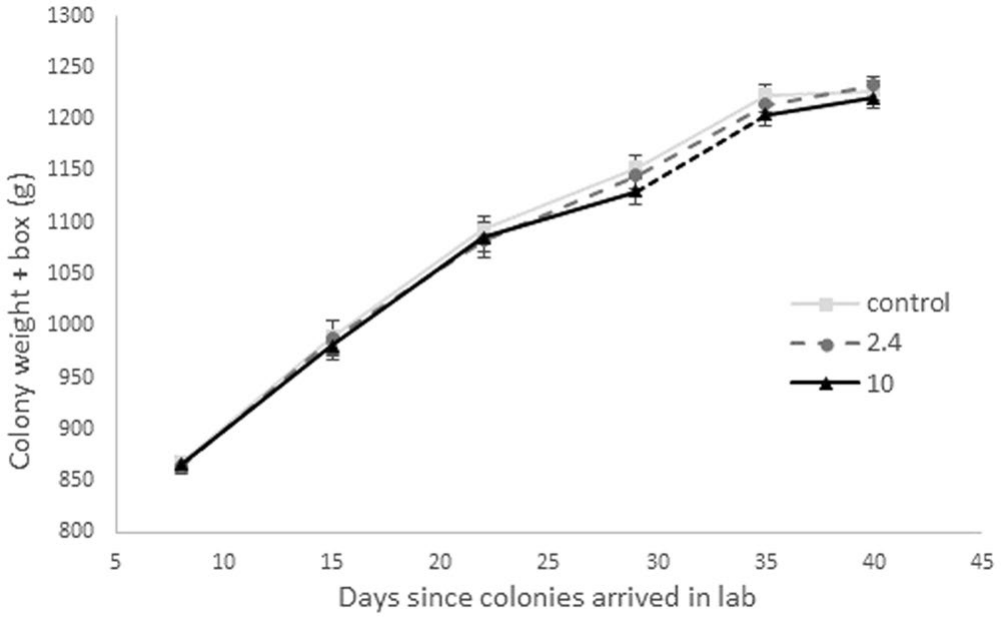
*Bombus terrestris* colony weight gain (g, including weight of outer box) over course of experiment from day 1 (2/4/2014) – day 40 (13/5/2014). N = eight colonies per treatment (24 colonies in total). Each colony was exposed to its respective treatment for a period of 26–27 days.

**Figure 2:**
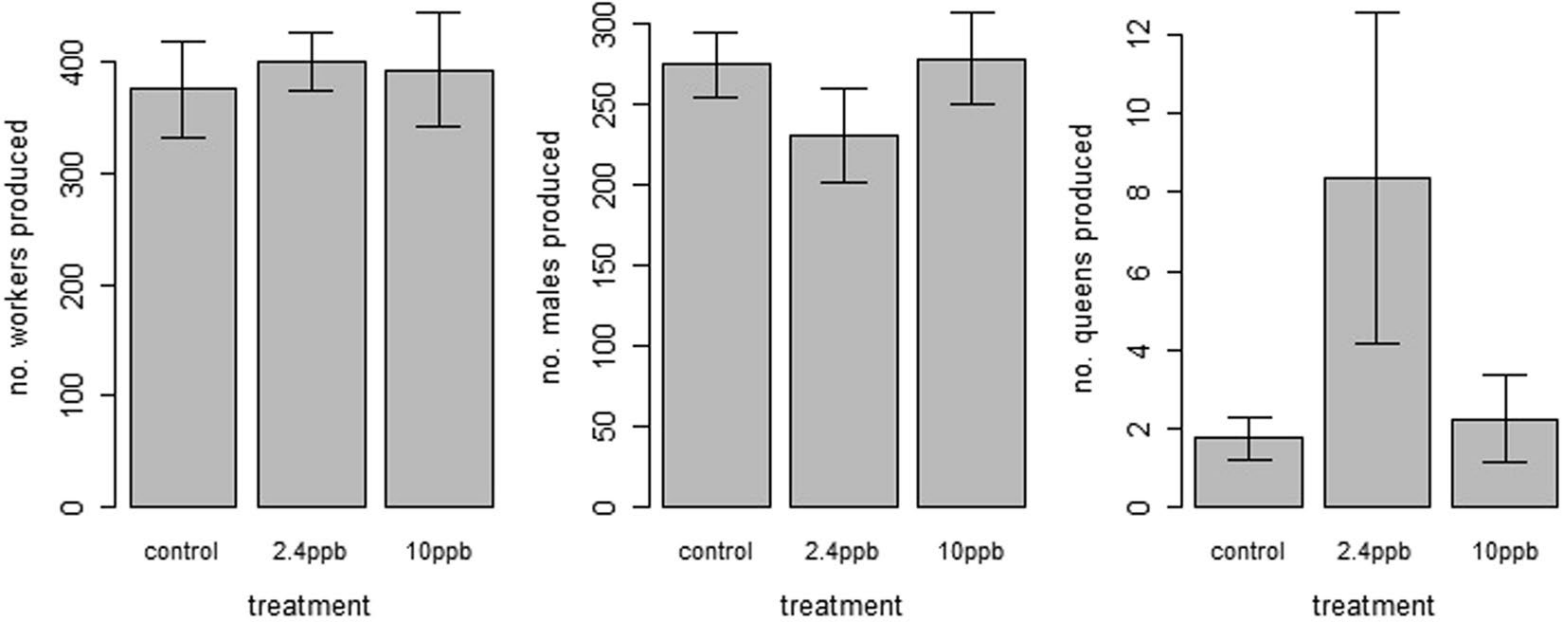
The mean number of workers, males and queens produced per *Bombus terrestris* colony, with colonies exposed to control, 2.4ppb thiamethoxam and 10ppb thiamethoxam. N = eight colonies per treatment (24 colonies in total), and error bars show standard error. No significant difference was found between any of the treatments in all three measures.

Exposure to pesticide did not influence the weight gained by colonies over the course of the experiment (F_2,21_ = 0.99, p = 0.39; Fig. 1), or the numbers of workers (F_2,21_ = 0.095, p = 0.9), males (F_2,21_ = 1.02, p = 0.38) or queens (KW test, χ2 = 1.14, df = 2, p = 0.57; Table 1; Fig. 2) produced. Although there was also no impact of treatment on weight of individual workers (F_2,21_ = 0.92, p = 0.41) or queens (F_2,21_ = 0.12, p = 0.89), there was a significant effect on the weight of males (F_2,21_ = 4.47, p = 0.02); males were significantly larger in colonies exposed to 2.4ppb pesticide than both controls and those exposed to 10ppb thiamethoxam (Table 1; Fig. S1). However, there was no effect of treatment on total biomass of any castes produced (workers: F_2,21_ = 0.73, p = 0.50, males: F_2,21_ = 0.03, p = 0.97, queens: F_2,21_ = 2.23, p = 0.13; Table 1; Fig. S2).

## Discussion

Our work shows that mature bumblebee colonies exposed chronically to field-realistic levels of thiamethoxam in a laboratory setting develop in a similar way to control colonies, with little impact of pesticide on colony growth or sexual production. The only measure that differed among treatment groups was the production of males; we found that colonies exposed to 2.4ppb thiamethoxam produced fewer males, but that the males produced were heavier than those from control or 10ppb thiamethoxam colonies. Taken together these patterns resulted in no differences across treatments in overall biomass of males produced. This suggests that colonies exposed to 2.4ppb thiamethoxam might differentially allocate resources, investing more resources into each male, but producing fewer overall. Although the difference was not significant, more colonies exposed to 2.4ppb pesticide produced queens (3 rather than 2) in this study, which could also be linked to differential investment by pesticide exposed colonies. However, as we found no significant impacts on other variables measured, there are unlikely to be strong implications for colony reproduction. Our work adds to the increasing body of research assessing the impacts of pesticide exposure on bumblebees.

### Laboratory tests

In this study we found no impacts of thiamethoxam exposure on bumblebee colony development under laboratory conditions. This is similar to findings reported by Laycock *et al*.^40^, who also observed no impacts of thiamethoxam at 16ppb or less on bumblebee feeding or fecundity in micro-colonies under laboratory conditions. However, other laboratory studies have found different patterns; for example Elston *et al*.^46^ reported reduced reproductive effort in bumblebee micro-colonies exposed to 10ppb thiamethoxam, and Fauser-Misslin *et al*.^39^ observed that exposure to pollen and nectar treated with 4ppb thiamethoxam and 1.5ppb clothianidin significantly reduced the reproductive success of whole colonies. Experimental colonies in our study were relatively large at the onset of pesticide exposure (containing an average of 99 workers), and it is likely that most of the workers, and some of the males, were already larvae or even pupae when pesticide exposure began. The colonies used in previous studies in which impacts of thiamethoxam on reproduction were found were exposed at an early stage of the colony cycle when, on average, only 10^39^ or 60^46^ workers were present. This suggests that pesticide effects may be more apparent at earlier stages of the colony cycle^34^, when individuals are also exposed to pesticide during larval development (when pollen consumption is much greater) and not only as adults. Oilseed rape (canola), one of the major flowering crops attractive to bees that is treated systemically with neonicotinoid pesticides, typically flowers in early summer when bumblebee colonies are likely to be at the start of development^49^. Although we did not find any impacts of treatment on colony weight (as a proxy for total colony size including brood), we did not specifically measure brood effects as colonies were large which prevented non-destructive counts of number of brood cells. Therefore, more work on the effects of pesticide exposure on a variety of colony developmental stages would be useful, as the severity of impacts on growth and reproduction are likely to be related to colony size at the time of exposure.

### Laboratory versus field studies

Our study was carried out in a laboratory setting, where bumblebees were fed nectar and pollen inside the nest box and, therefore, did not have to work hard to forage or provision their colonies. Under field and semi-field conditions, bumblebees are faced with more challenging environments and need to forage (at least partially) for their own food from real flowers. As exposure to both imidacloprid^33, 50^ or thiamethoxam^42–45^ can affect bumblebee learning and foraging ability, it is unsurprising that effects on colony development are likely to be more severe under field conditions. A second point is that in the field bumblebees are also exposed to variable amounts of mixtures of pesticides in the nectar and pollen of crops and wild plants^18, 21, 51^. Although it is hard to replicate this level or variability in the lab setting, it is important to understand the effects of consistent amounts of single pesticides before examining variability. Therefore, we would suggest running field studies in conjunction with laboratory-based experiments when investigating pesticide impacts on bumblebee reproduction to ensure results are representative of the wider environment.

### Impacts of different neonicotinoids

Impacts of thiamethoxam exposure on colony development under laboratory conditions are variable, but reported impacts of imidacloprid on bumblebee colony development and reproduction at field realistic levels are more consistent, with dose-dependent reductions in fecundity in micro-colonies^36^ and reduced colony development of entire colonies^52^. In semi-field studies, although thiamethoxam exposure led to changes in foraging patterns and reduced pollen collection, impacts on development in a small number of colonies were not recorded^44^, and even an increase in colony weight has been reported (although this study lacks appropriate replication^53^). On the other hand, colonies exposed to imidacloprid showed changes in foraging patterns and reduced pollen collection, and subsequent deficits in colony development^34^, colony growth^37^ and queen production^35^. With two experiments using a similar setup, bumblebee micro-colonies exposed to imidacloprid in the lab showed a dose-dependent decline in fecundity, starting at field-realistic levels, which was attributed to reduced feeding on both syrup and pollen^36^, but no effects on feeding or micro-colony development at field-realistic levels of thiamethoxam exposure^40^. Studies investigating clothianidin impacts have found: 1) fewer adult workers and sexuals produced in colonies exposed to 5ppb clothianidin^41^, 2) reduced growth and reproduction in bumblebee colonies placed beside clothianidin treated oilseed rape fields in a fully replicated field experiment^31^ and 3) no effect on bumblebee reproduction (although this study lacked appropriate replication^54^). Moffat *et al*.^55^ found compound specific effects of different neonicotinoids on different variables; for example, exposure to 2.5ppb imidacloprid and thiamethoxam (but not clothianidin) decreased colony strength, whereas increased queen production was only found in colonies treated with clothianidin. Together, these studies support the view that exposure to various neonicotinoids result in differential impacts for bumblebees. However, the majority of these comparisons are circumstantial, and more work is needed to directly compare the effects of different neonicotinoid types on bumblebees using common methodological approaches and exposure levels.

## Conclusion

One of the major concerns about the impacts of pesticides on bees is that the pollination services they deliver may be affected. Although we find no impacts of thiamethoxam on the growth of mature bumblebee colonies in this study, previous work has found impacts on queen reproductive development^48^ and ability to found colonies^47^, and that worker bees exposed to thiamethoxam show reduced learning ability, reduced pollen collection, and differential visitation of flowers^42, 45^. Therefore, although thiamethoxam may not cause impacts on colonies under laboratory conditions, it could have different impacts in a field setting where the ability to forage and collect pollen are critical for normal colony development^56^, and where bees are often exposed to multiple pesticides simultaneously^21^. Bees with impaired learning and/or pollen collecting ability are unlikely to be delivering the same level of pollination services to crops and wild plants. Therefore, although impacts on colony development may not be evident, pollination service delivery could be affected by changes in individual behaviour related to thiamethoxam exposure rather than via impacts at the colony level.

Our work shows that chronic exposure to a neonicotinoid pesticide in a laboratory setting has little impact on the growth of mature bumblebee colonies. A comparison of our work with other published studies leads us to make a number of suggestions for future research directions and pesticide testing. Firstly, pesticide effects should be examined at multiple stages of the colony cycle to ensure any differences between effects on mature and developing colonies are understood^47, 48, 57^, as they may not be similar. Secondly, pesticide testing should be carried out under both laboratory and field conditions, because bees under field conditions may be more susceptible to pesticide effects than those in the laboratory. Lastly, there is a need to directly compare different neonicotinoid pesticides, using common methodologies, as effects of thiamethoxam may be different to other neonicotinoids (e.g. imidacloprid).

## Materials and Methods

Insecticide solutions of 2.4 and 10ppb thiamethoxam used in subsequent experimental procedures were prepared as follows: an initial stock solution was created by dissolving 100 mg thiamethoxam (PESTANAL, Analytical Standard, Sigma Aldrich) in 100 ml acetone (1 mg/ml). We then added 10 µg or 2.4 µg of this solution to a litre of 40% sucrose, to create solutions of 10ppb and 2.4ppb thiamethoxam, respectively (these concentrations are calculated on a volume/ volume (v/v) basis; on a weight/ weight (w/w) basis solutions would be 2ppb and 8.5ppb, respectively). These concentrations were chosen as field-realistic; the lower concentration (2.4ppb) was measured in nectar pots of bumblebee colonies foraging in agricultural areas in the UK^12^ and in pollen collected by honeybees in France^38^, while the higher concentration (10ppb) is within the range that has been measured in the nectar and pollen of a variety of treated crops and wild plants^19, 58, 59^, and appreciably below the mean levels found in pollen collected by bumblebees in UK rural areas^21^. We also made a control solution by repeating the process outlined above but using an aliquot of 10ppb acetone only (i.e. no pesticide).

Twenty-four *Bombus terrestris audax* colonies were obtained from Biobest (Westerlo, Belgium) at the start of April 2014, each containing a queen and an average of 99 workers (range 57–133). On arrival, colonies were weighed to estimate overall colony size (without either the sucrose feeder reservoir and outer cardboard box), and then assigned sequentially to one of three treatment groups (10ppb thiamethoxam, 2.4ppb thiamethoxam and control) based on decreasing mass (but randomly assigned within block). Each day, three colonies (one from each treatment) began treatment, until after 7 days all colonies were receiving their respective sucrose solutions (eight control colonies, eight colonies exposed to 2.4ppb thiamethoxam, and eight colonies exposed to 10ppb thiamethoxam). Each colony was exposed to its respective treatment for a period of 26–27 days, and all sucrose solutions were actively consumed. Colonies were weighed weekly over the entire study period, to estimate colony growth. All colonies were given a large gravity feeder containing their treated solution each day, and were fed equal amounts of untreated, defrosted, honeybee-collected pollen.

After 26–27 days of exposure to their respective treatments, treatment of colonies stopped. All colonies were then fed untreated sucrose until 13^th^ May (40 days since colonies had arrived from the supplier), when all colonies were frozen at −20 °C. Subsequently, colonies were dissected and the numbers of workers, males and queens (gynes) produced were counted. Thirty workers, 30 males, and all queens were dried in a drying oven at 60 °C for 3 days, and then weighed to give the dry mass of each individual. This information was used to a) calculate an average dry mass (weight) of individuals of each caste in each colony, and b) to estimate the total dry mass of each caste produced^60^.

We tested for differences in weight gain, average dry mass of individuals of each caste (workers, males and queens), and total biomass of each caste using analysis of variance. Differences in the numbers of males and workers produced were tested using generalized linear models with Poisson distribution specified for counts, and a Kruskal-Wallis test for numbers of queens produced as data were non-normally distributed. All models were verified by inspecting distribution of residuals, and all analyses were carried out in the stats package in R v 3.1.10^61^.

### Data availability statement

All data are available in the supplementary information

## Electronic supplementary material

Supplemetary info

## References

[1] Corbet SA, Williams IH, Osborne JL (1991). Bees and the pollination of crops and wild flowers in the European community. Bee World.

[2] Button L, Elle E (2014). Wild bumble bees reduce pollination deficits in a crop mostly visited by managed honey bees. Agriculture, Ecosystems & Environment.

[3] Kleijn D (2015). Delivery of crop pollination services is an insufficient argument for wild pollinator conservation. Nature Communications.

[4] Williams PH, Osborne JL (2009). Bumblebee vulnerability and conservation world-wide. Apidologie.

[5] Grixti JC, Wong LT, Cameron SA, Favret C (2009). Decline of bumble bees (*Bombus*) in the North American Midwest. Biological Conservation.

[6] Dupont YL, Damgaard C, Simonsen V (2011). Quantitative historical change in bumblebee (*Bombus* spp.) assemblages of red clover fields. PLoS One.

[7] Bommarco R, Lundin O, Smith HG, Rundlöf M (2011). Drastic historic shifts in bumble-bee community composition in Sweden. Proc. B.

[8] Cameron SA (2011). Patterns of widespread decline in North American bumble bees. PNAS.

[9] Williams P (1986). Environmental change and the distributions of British bumble bees (*Bombus* Latr.). Bee World.

[10] Williams PH (1982). The distribution and decline of British Bumble Bees (*Bombus* Latr.). Journal of Apicultural Research.

[11] Goulson D (2013). An overview of the environmental risks posed by neonicotinoid insecticides. Journal of Applied Ecology.

[12] Thompson, H. *et al*. Effects of neonicotinoid seed treatments on bumble bee colonies under field conditions. York, UK, Food and Environment Research Agency, 2013.

[13] Pohorecka K (2012). Residues of neonicotinoid insecticides in bee collected plant materials from oilseed rape crops and their effect on bee colonies. Journal of Apicultural Science.

[14] Cutler GC, Scott-Dupree CD (2014). A field study examining the effects of exposure to neonicotinoid seed-treated corn on commercial bumble bee colonies. Ecotoxicology.

[15] Jones A, Harrington P, Turnbull G (2014). Neonicotinoid concentrations in arable soils after seed treatment applications in preceding years. Pest Management Science.

[16] Hladik ML, Kolpin DW, Kuivila KM (2014). Widespread occurrence of neonicotinoid insecticides in streams in a high corn and soybean producing region, USA. Environmental Pollution.

[17] Samson-Robert O, Labrie G, Chagnon M, Fournier V (2014). Neonicotinoid-contaminated puddles of water represent a risk of intoxication for honey bees. PLoS One.

[18] Krupke CH, Hunt GJ, Eitzer BD, Andino G, Given K (2012). Multiple routes of pesticide exposure for honey bees living near agricultural fields. PLoS One.

[19] Stewart SD (2014). Potential exposure of pollinators to neonicotinoid insecticides from the use of insecticide seed treatments in the mid-southern United States. Environmental Science & Technology.

[20] Botías C (2015). Neonicotinoid residues in wildflowers, a potential route of chronic exposure for bees. Environmental Science & Technology.

[21] David A (2016). Widespread contamination of wildflower and bee-collected pollen with complex mixtures of neonicotinoids and fungicides commonly applied to crops. Environment International.

[22] Long EY, Krupke CH (2016). Non-cultivated plants present a season-long route of pesticide exposure for honey bees. Nature Communications.

[23] Desneux N, Decourtye A, Delpuech J-M (2007). The sublethal effects of pesticides on beneficial arthropods. Annual Review of Entomology.

[24] Godfray HCJ (2014). A restatement of the natural science evidence base concerning neonicotinoid insecticides and insect pollinators. Proceedings of the Royal Society B- Biological Sciences.

[25] Godfray HCJ (2015). A restatement of recent advances in the natural science evidence base concerning neonicotinoid insecticides and insect pollinators. Proceedings of the Royal Society B- Biological Sciences.

[26] Lundin O, Rundlöf M, Smith HG, Fries I, Bommarco R (2015). Neonicotinoid insecticides and their impacts on bees: a systematic review of research approaches and identification of knowledge gaps. PLoS One.

[27] Cabrera AR (2016). Initial recommendations for higher-tier risk assessment protocols for bumble bees, *Bombus* spp. (Hymenoptera: Apidae). Integrated Environmental Assessment and Management.

[28] Arena M, Sgolastra F (2014). A meta-analysis comparing the sensitivity of bees to pesticides. Ecotoxicology.

[29] Cresswell JE (2012). Differential sensitivity of honey bees and bumble bees to a dietary insecticide (imidacloprid). Zoology.

[30] Piiroinen S, Goulson D (2016). Chronic neonicotinoid pesticide exposure and parasite stress differentially affects learning in honeybees and bumblebees. Proceedings of the Royal Society B- Biological Sciences.

[31] Rundlöf M (2015). Seed coating with a neonicotinoid insecticide negatively affects wild bees. Nature.

[32] Woodcock BA (2017). Country-specific effects of neonicotinoid pesticides on honey bees and wild bees. Science.

[33] Feltham H, Park K, Goulson D (2014). Field realistic doses of pesticide imidacloprid reduce bumblebee pollen foraging efficiency. Ecotoxicology.

[34] Gill RJ, Ramos-Rodriguez O, Raine NE (2012). Combined pesticide exposure severely affects individual- and colony-level traits in bees. Nature.

[35] Whitehorn PR, O’Connor S, Wackers FL, Goulson D (2012). Neonicotinoid pesticide reduces bumble bee colony growth and queen production. Science.

[36] Laycock I, Lenthall KM, Barratt AT, Cresswell JE (2012). Effects of imidacloprid, a neonicotinoid pesticide, on reproduction in worker bumble bees (*Bombus terrestris*). Ecotoxicology.

[37] Moffat C (2015). Chronic exposure to neonicotinoids increases neuronal vulnerability to mitochondrial dysfunction in the bumblebee (*Bombus terrestris*). The FASEB Journal.

[38] Pilling E, Campbell P, Coulson M, Ruddle N, Tornier I (2013). A four-year field program investigating long-term effects of repeated exposure of honey bee colonies to flowering crops treated with thiamethoxam. PLoS One.

[39] Fauser-Misslin A, Sadd B, Neumann P, Sandrock C (2014). Influence of combined pesticide and parasite exposure on bumblebee colony traits in the laboratory. Journal of Applied Ecology.

[40] Laycock I, Cotterell KC, O’Shea-Wheller TA, Cresswell JE (2014). Effects of the neonicotinoid pesticide thiamethoxam at field-realistic levels on microcolonies of *Bombus terrestris* worker bumble bees. Ecotoxicology and Environmental Safety.

[41] Arce, A. N. *et al*. Impact of controlled neonicotinoid exposure on bumblebees in a realistic field setting. *Journal of Applied Ecology***online early**, doi:10.1111/1365-2664.12792 (2016).

[42] Stanley DA (2015). Neonicotinoid pesticide exposure impairs crop pollination services provided by bumblebees. Nature.

[43] Stanley DA, Raine NE (2016). Chronic exposure to a neonicotinoid pesticide alters the interactions between bumblebees and wild plants. Functional Ecology.

[44] Stanley DA, Russell AL, Morrison SJ, Rogers C, Raine NE (2016). Investigating the impacts of field-realistic exposure to a neonicotinoid pesticide on bumblebee foraging, homing ability and colony growth. Journal of Applied Ecology.

[45] Stanley DA, Smith KE, Raine NE (2015). Bumblebee learning and memory is impaired by chronic exposure to a neonicotinoid pesticide. Scientific Reports.

[46] Elston C, Thompson H, Walters KA (2013). Sub-lethal effects of thiamethoxam, a neonicotinoid pesticide, and propiconazole, a DMI fungicide, on colony initiation in bumblebee (*Bombus terrestris*) micro-colonies. Apidologie.

[47] Baron, G. L., Jansen, V. A. A., Brown, M. J. F., & Raine, N. E. Pesticide reduces bumblebee colony establishment and increases probability of population extinction. *Nature Ecology & Evolution* (2017 in press). 10.1038/s41559-017-0260-1 PMC6485633 29046553

[48] Baron, G. L., Raine, N. E. & Brown, M. J. F. General and species-specific impacts of a neonicotinoid insecticide on the ovary development and feeding of wild bumblebee queens. *Proceedings of the Royal Society B: Biological Sciences* **284** (2017). 10.1098/rspb.2017.0123 PMC5443941 28469019

[49] Stanley DA, Gunning D, Stout JC (2013). Pollinators and pollination of oilseed rape crops (*Brassica napus* L.) in Ireland: ecological and economic incentives for pollinator conservation. Journal of Insect Conservation.

[50] Gill RJ, Raine NE (2014). Chronic impairment of bumblebee natural foraging behaviour induced by sublethal pesticide exposure. Functional Ecology.

[51] Botías C, David A, Hill EM, Goulson D (2017). Quantifying exposure of wild bumblebees to mixtures of agrochemicals in agricultural and urban landscapes. Environmental Pollution.

[52] Bryden J, Gill RJ, Mitton RAA, Raine NE, Jansen VAA (2013). Chronic sublethal stress causes bee colony failure. Ecology Letters.

[53] Thompson H (2016). Monitoring the effects of thiamethoxam applied as a seed treatment to winter oilseed rape on the development of bumblebee (*Bombus terrestris*) colonies. Pest Management Science.

[54] Sterk G, Peters B, Gao Z, Zumkier U (2016). Large-scale monitoring of effects of clothianidin-dressed OSR seeds on pollinating insects in Northern Germany: effects on large earth bumble bees (*Bombus terrestris*). Ecotoxicology.

[55] Moffat C (2016). Neonicotinoids target distinct nicotinic acetylcholinase receptors and neurons, leading to differential risks to bumblebees. Scientific Reports.

[56] Pelletier L, McNeil JN (2003). The effect of food supplementation on reproductive success in bumblebee field colonies. Oikos.

[57] Fauser A, Sandrock C, Neumann P, Sadd B (2017). Neonicotinoids override a parasite exposure impact onhibernation success of a key bumblebee pollinator. Ecological Entomology.

[58] Dively GP, Kamel A (2012). Insecticide residues in pollen and nectar of a cucurbit crop and their potential exposure to pollinators. Journal of Agricultural and Food Chemistry.

[59] Castle SJ, Byrne FJ, Bi JL, Toscano NC (2005). Spatial and temporal distribution of imidacloprid and thiamethoxam in citrus and impact on *Homalodisca coagulata* populations. Pest Management Science.

[60] Baron GL, Raine NE, Brown MJF (2014). Impact of chronic exposure to a pyrethroid pesticide on bumblebees and interactions with a trypanosome parasite. Journal of Applied Ecology.

[61] R: A language and environment for statistical computing. (R Foundation for Statistical Computing, Vienna, Austria. http://www.R-project.org, 2014).

