# Supplementary material for: Bumblebee colony development following chronic exposure to field-realistic levels of the neonicotinoid pesticide thiamethoxam under laboratory conditions
Source: Sci Rep. 2017 Aug 14;7:8005. doi: 10.1038/s41598-017-08752-x (PMC5556064; doi:10.1038/s41598-017-08752-x)
Supplement: Supplementary file 1 — Supplemetary info [file 41598_2017_8752_MOESM1_ESM.pdf]

Bumblebee colony development following chronic exposure to field-realistic levels of the neonicotinoid pesticide thiamethoxam under laboratory conditions

SUPPLEMENTARY INFORMATION

Dara A. Stanley[1,2*] & Nigel E. Raine[1,3]

[1] School of Biological Sciences, Royal Holloway University of London, Egham, TW20 0EX, UK; [2] Botany and Plant Science, School of Natural Sciences and Ryan Institute, National University of Ireland Galway, Ireland; [3] School of Environmental Sciences, University of Guelph, Guelph, Ontario, N1G 2W1, Canada

*corresponding author: dara.stanley@nuigalway.ie

Table S1. Data collected from the 24 bumblebee colonies exposed to control, 2.4ppb thiamethoxam and 10ppb thiamethoxam solutions. Total biomass measures (g) come from dry mass weights collected from 30 individuals, but scaled to the number of individuals of the caste per colony.

Colony no.	No. workers on arrival	Treatment	Date treatment began	Weight at start of experiment (g)	Weight at end of experiment (g)	Date treatment stopped	Date colonies were frozen	No. males	No. workers	No. queens	Total queen biomass	Total worker biomass	Total male biomass
2	97	control	04/04/14	567.2	868.7	01/05/14	13/05/14	232	384	3	0.56	17.69	18.37
8	105	control	07/04/14	559.4	879.8	03/05/14	13/05/14	367	239	5	0.74	17.23	31.98
12	85	control	08/04/14	544.6	830.7	04/05/14	13/05/14	338	146	1	0.24	6.17	30.76
17	105	control	11/04/14	516	863.4	07/05/14	13/05/14	197	405	1	0.28	19.44	14.68
20	82	control	09/04/14	542.4	918.9	05/05/14	13/05/14	238	428	1	0.28	23.08	19.27
23	100	control	06/04/14	560.6	927.2	02/05/14	13/05/14	264	511	1	0.32	28.68	24.94
24	102	control	10/04/14	532.2	881.1	06/05/14	13/05/14	273	404	1	0.28	19.30	22.76
25	73	control	03/04/14	570.6	915.7	30/04/14	13/05/14	288	483	1	0.30	23.86	21.19
1	124	10ppb	10/04/14	533.2	870.7	06/05/14	13/05/14	177	527	1	0.27	23.05	17.33
4	132	10ppb	03/04/14	575	933.3	30/04/14	13/05/14	231	606	1	0.21	27.96	20.09
9	106	10ppb	06/04/14	561.4	882	02/05/14	13/05/14	319	399	1	0.31	20.19	26.92
10	113	10ppb	07/04/14	554.4	882.9	03/05/14	13/05/14	188	500	10	1.70	23.53	16.93
11	80	10ppb	08/04/14	546.8	896.9	04/05/14	13/05/14	378	330	1	0.24	18.49	33.62
15	117	10ppb	11/04/14	528	812.6	07/05/14	13/05/14	397	161	1	0.26	9.58	35.16
16	94	10ppb	09/04/14	540.2	845	05/05/14	13/05/14	281	269	1	0.26	15.53	24.81
19	100	10ppb	04/04/14	565	860.3	01/05/14	13/05/14	257	350	2	0.43	17.99	16.75
3	104	2.4ppb	04/04/14	566.4	878.8	01/05/14	13/05/14	138	431	1	0.28	23.09	17.26
5	90	2.4ppb	08/04/14	544.8	972.2	04/05/14	13/05/14	189	471	1	0.28	22.14	24.45
6	101	2.4ppb	09/04/14	541.6	858.1	05/05/14	13/05/14	250	440	1	0.23	20.80	17.77
13	133	2.4ppb	03/04/14	576.8	873.4	30/04/14	13/05/14	306	367	14	3.39	19.07	39.32
14	86	2.4ppb	11/04/14	520.8	942.5	07/05/14	13/05/14	199	491	1	0.29	26.35	15.53
18	102	2.4ppb	07/04/14	552.6	881.5	03/05/14	13/05/14	369	277	1	0.27	19.70	30.98
21	89	2.4ppb	06/04/14	560.4	883	02/05/14	13/05/14	132	385	34	8.03	21.69	14.37
22	57	2.4ppb	10/04/14	531	896.8	06/05/14	13/05/14	262	338	14	2.45	24.69	28.59

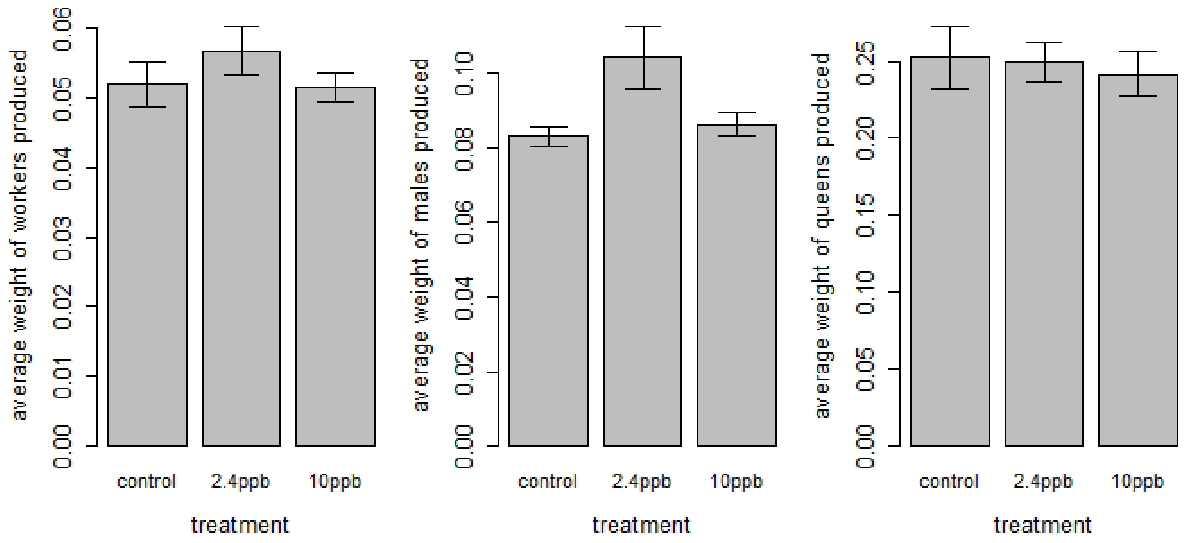

Figure S1. The average weight (g) of workers, males and queens produced per colony, with colonies exposed to control, 2.4ppb thiamethoxam and 10ppb thiamethoxam. N=8 colonies per treatment (24 colonies in total). Error bars show standard error.

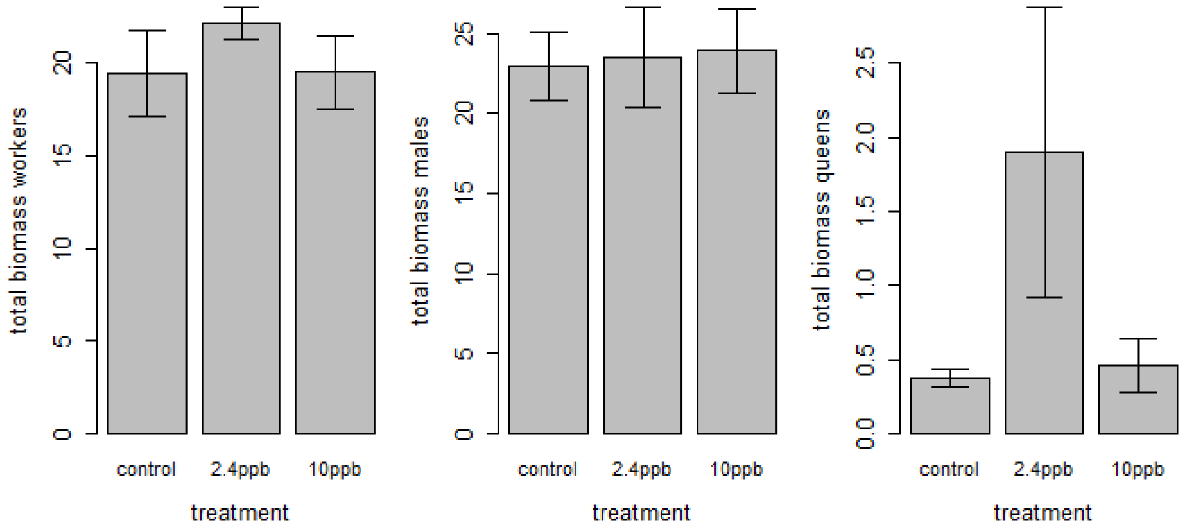

Figure S2. The total biomass (g) of workers, males and queens produced per colony, with colonies exposed to control, 2.4ppb thiamethoxam and 10ppb thiamethoxam. N=8 colonies per treatment (24 colonies in total). Error bars show standard error.
